# Effective control of two genotypes of *Phytophthora infestans* in the field by three oxathiapiprolin fungicidal mixtures

**DOI:** 10.1371/journal.pone.0258280

**Published:** 2021-10-08

**Authors:** Yigal Cohen, Avia E. Rubin, Mariana Galperin

**Affiliations:** Faculty of Life Sciences, Bar Ilan University, Ramat Gan, Israel; Lovely Professional University, INDIA

## Abstract

In two field experiments, performed in 2020 and 2021, potato Nicola plants were sprayed once with three (Exp. 1) or two (Exp. 2) doses of Zorvec Vinabel (oxathiapiprolin+ zoxamide = ZZ), Zorvec Encantia (oxathiapiprolin+ famoxadone = ZF), Zorvec Endavia (oxathiapiprolin+ benthiavalicarb = ZE), Infinito (= INF) or Mefenoxam (= MFX) and thereafter inoculated with genotype 23A1 or 36A2 of *Phytophthora infestans*. Disease development was recorded at periodic intervals for a month. In both experiments, Zorvec mixtures were significantly more effective in suppressing the disease than INF or MFX. They delayed the onset of the disease and its progress, regardless the genotype used for inoculation. Among the three Zorvec mixtures, ZZ was least effective and ZE most effective. Sensitivity monitoring assays revealed zero mutants of *P*. *infestans* resistant to oxathiapiprolin. The data confirmed good efficacy of Zorvec mixtures, especially ZE, in field-grown potato crops as evident by the very effective control of late blight for one month.

## Introduction

Late blight caused by the oomycete *Phytophthora infestans* (Mont.) De Barry remains one of the most destructive and economically important diseases of potato and tomato world-wide including Israel. Control options include the use of host resistance, disease risk forecasting and optimal fungicide use, alone and in combination. However, in practice, management typically relies on a prophylactic fungicide program at 5-7day intervals to minimize the potentially severe impact of a field outbreak. The efficacy of the fungicide program depends on the genotype of the pathogen, its initial inoculum level, and the conduciveness of the environment to infection [1].

Recently introduced fungicides include oxathiapiprolin (2017), oxathiapiprolin+ famoxadone (2018), oxathiapiprolin+ amisulbrom (2018), benthiavalicarb (2018), oxathiapiprolin+ benthiavalicarb (2019) and fluoxapiprolin (2020). Oxathiapiprolin and fluoxapiprolin are piperidinyl thiazole isoxazoline fungicides (FRAC code 49, previously U15) that target the oxysterol binding proteins in oomycete cells [2–4]. Oxathiapiprolin is extremely active against plant pathogenic oomycetes except Pythium (see literature cited by [5]). It shows translaminar and acropetally systemic movements [5–7]. Oxathiapiprolin is highly effective when applied to the foliage [5], the root system [8], the seeds [9] or to the soil [10].

Oxathiapiprolin is a single site inhibitor (OBSPI, oxysterol binding protein inhibitor). Resistance risk assumed to be medium to high [(single site and therefore resistance management required (https://www.frac.info)]. Indeed, mutants of *Phytophthora capsici* resistant to oxathiapiprolin were produced in the laboratory [11] and recently isolated from field grown cucumber fruits [12]. Field resistance to oxathiapiprolin occurred in *P*. *infestans* in Indonesia and in *Plasmopara viticola* in Europe (https://www.frac.info).

To reduce the risk of resistance development, FRAC recommends making no more than four applications or maximum 33% of the total period of protection needed per crop, whichever is more restrictive. Where the total number of fungicide applications targeting oomycetes is less than three, apply no more than one application of an OSBPI product. Applications of OSBPI-containing products are to be made no more than three times in sequence before applying a fungicide with a different mode of action. In areas where the agronomic risk is very high (e.g. continuous potato or cucurbit cropping) and resistance has already been reported, further restrictions to the number of consecutive applications are recommended. Applications of OSBPI products can be made in alternation with a fungicide with a different mode of action (https://www.frac.info).

In both autumn and spring growing seasons of potato in Israel, the environmental conditions for late blight outbreaks and development are optimal. Tubers seeds are produced locally for sowing in the autumn, but for the spring season tubers seeds are imported from Europe. This import involves an annual import of new genotypes of *P*. *infestans* from Europe which changes the population structure of the pathogen in the country. Our long-standing monitoring surveys [13] showed that the major genotype that prevails in the country in the past 12 years is 23A1. Other genotypes occurred in the country on a temporary basis. Thus, US-7 like occurred during 2008–2015; 13A2 -during 2016–2017 and 36A2 from 2018 to present. Genotypes differed in their SSR profile, mating type, virulence factors, aggressiveness, and sensitivity to fungicides. Although the major weapons against late blight are fungicides, no study has been done to compare the efficacy of different classes of fungicides against different genotypes of the pathogen under field conditions. Such a comparative study is reported here.

## Materials and methods

### Site location

The field experiments reported here were conducted at BIU Farm located on campus of Bar Ilan University, Israel (32° 04’2.40” N; 34°50’19.79” E), 5 km west of Tel-Aviv.

### Weather

Air temperature, relative humidity and rain fall were recorded every 10 minutes in a weather station located on campus. The number of hours per day in which the relative humidity was ≥90% (indicating on leaf wetness) when air temperature was 9–21°C was recorded.

### Plants

Seed potato tuber *cv* Nicola (imported from Holland) were a gift from U. Zig, Yaham Ltd, Israel. In Experiment 1, five tubers were sown on 22.10.2020 in each of 110 polystyrene containers (120x60x22 cm) filled with peat: perlite mixture (10:1, v/v). Containers were arranged in 3 rows in a 50x6 m net house at BIU Farm. Four replicate containers were used for each of 4 fungicides/ 3 doses/ 2 genotypes, totaling 24 treatments in 96 containers. Fourteen containers served as controls. The east half of the net house was inoculated with genotype 23A1 of *P*. *infestans* while the west half was inoculated with genotype 36A2 (see below). In Experiment 2, four tubers (as above) were sown on 22.12.2020 in each of 92 polystyrene containers (80x60x22 cm) filled with peat: perlite mixture (10:1, v/v). Containers were arranged in 2 rows in a 50x6 m net house at BIU Farm. Four replicate containers were used for each of 5 fungicides/ 2 doses/ 2 genotypes, totaling 20 treatments in 80 containers. Twelve containers served as controls. The left (southern) row was inoculated with genotype 23A1 while the right (northern) row was inoculated with genotype 36A2 (see below).

### Fungicides

Four fungicides were used in Experiment 1: Infinito 68.75% (= INF, fluopicolide 62.5 g/L+ propamocarb 625 g/L); Zorvec Vinabel 32.3% (= ZZ, oxathiapiprolin 39 g/L + zoxamide 284 g/L); Zorvec Encantia 30% (= ZF, oxathiapiprolin 30 g/L + famoxadone 300 g/L); Zorvec Endavia 10% (= ZE, oxathiapiprolin 30 g/L + benthiavalicarb 70 g/L). Three dose concentrations of each product were used: 0.25, 0.5 and 1% v/v. Plants were sprayed only once, on 22.11.2020, at 30 days after sowing, when reached ~20 cm height and had 12 true leaves. Spray was applied to the upper leaf surfaces by a hand sprayer. About 200 ml of each fungicide suspension were applied to 40 plants in 8 containers.

Five fungicides were used in Experiment 2: INF, ZZ, ZF, ZE and MFX (mefenoxam 48%). Two dose concentrations of each product were used: 0.1 and 1% v/v. Plants were sprayed only once, on 25.1.2021, at 31 days after sowing, when had reached ~20 cm height and had 12 true leaves. Spray was applied to the upper leaf surfaces by a hand sprayer. About 160 ml of each fungicide suspension were applied to 32 plants in 8 containers.

### Pathogen

Fifteen isolates of *P*. *infestans* genotype (lineage) 23A1 and 15 isolates of *P*. *infestans* genotype (lineage) 36A2 were used for inoculation. Isolates were collected from potato fields in Western Negev, Israel during the spring season of 2020. Genotype analysis was conducted by D. L. Cooke of Hutton Institute, Dundee, Scotland. Their virulence to potato was determined by inoculating potato lines carrying R genes 1–11 [14]. No isolate was compatible with R gene 8. Most 23A1 isolates were incompatible with R genes 5 and 10 whereas most 36A2 isolates were incompatible with R genes 2 and 6. Most 23A1 isolates were resistant to mefenoxam, whereas most 36A2 isolates were sensitive to mefenoxam. The isolates were maintained and propagated in growth chambers at 20°C by repeated inoculation of detached tomato leaves. For inoculation in the field, fresh, 12h-old sporangia were used. The sporangia were collected at 6 dpi from heavily sporulating, detached tomato leaves and kept on ice until used (1-2h).

### Inoculation

In both experiments, plants were inoculated at 6–7 hours after fungicide application (at 17 pm). One liter of sporangial suspension (2000 sporangia per ml), made of fifteen isolates of genotype 23A1 or fifteen isolates of genotype 36A2, was used for inoculations. In Experiment 1, plants in the 55 eastern containers were spray-inoculated with 1L of sporangial mixture of genotype 23A1 while the 55 western containers were inoculated with 1L of sporangial mixture of genotype 36A2. In Experiment 2, plants in the 46 southern containers were spray-inoculated with 1L of sporangial mixture of genotype 23A1 while the 46 northern containers were inoculated with 1L of sporangial mixture of genotype 36A2. The inoculated plants were covered with plastic sheets until 8 am of the following day to provide high humidity required for successful infection. In Experiment 2, the inoculation procedure was repeated on 3.2.2021 due to the poor infection obtained after the first inoculation.

### Disease evaluation

In Experiment 1, % blighted foliage area was visually estimated in plants in each container starting on 5 days post inoculation (dpi). Thirteen such records were taken, every 2 days until 28 dpi, when the control plots were fully devastated of late blight. In Experiment 2, % blighted foliage area was estimated ten times, starting at 8 days post (first) inoculation, every 2–3 days, until 34 dpi, when the control plots were almost fully devastated of late blight.

### Resistance monitoring

Infected leaflets (1–5 leaflets per treatment, depending on their frequency) were collected at 14 and 28 dpi in Exp.1 and at 27 and 34 dpi in Exp.2. Assays were performed as previously described [6] with slight modifications. Briefly, infected leaves were placed in moisten Petri dished overnight to induce sporulation. The sporangia were collected into ice-cold distilled water and drop inoculated onto detached tomato leaves (cultivar Baby) previously sprayed with oxathiapiprolin of 0, 0.0001, 0.001, 0.01 or 0.1 ppm ai. Each tomato leaflet (n = 3) was inoculated with ten 10μl of sporangia suspension, 200 sporangia per droplet. Plates were incubated at 18°C in the dark overnight and thereafter in a growth chamber at 20C with 14h light a day (100μE.m^-1^.s^-1^). The number of sporulating lesions developed on the inoculated leaves was counted at 7 dpi.

### Data analysis

Disease records were taken at 2–3 days interval from 4 replicate containers (except ZF 0.5%, two containers in Exp 1). Mean % blighted foliage area and standard deviation of the mean were calculated for each treatment at each scoring day. t-test at α = 0.05 was used to determine if differences between treatments (fungicides, doses, genotypes) are significant. AUDPC (area under disease progress curve) values were calculated for comparison purposes.

## Results

### Experiment 1

#### Weather

**Fig 1:**
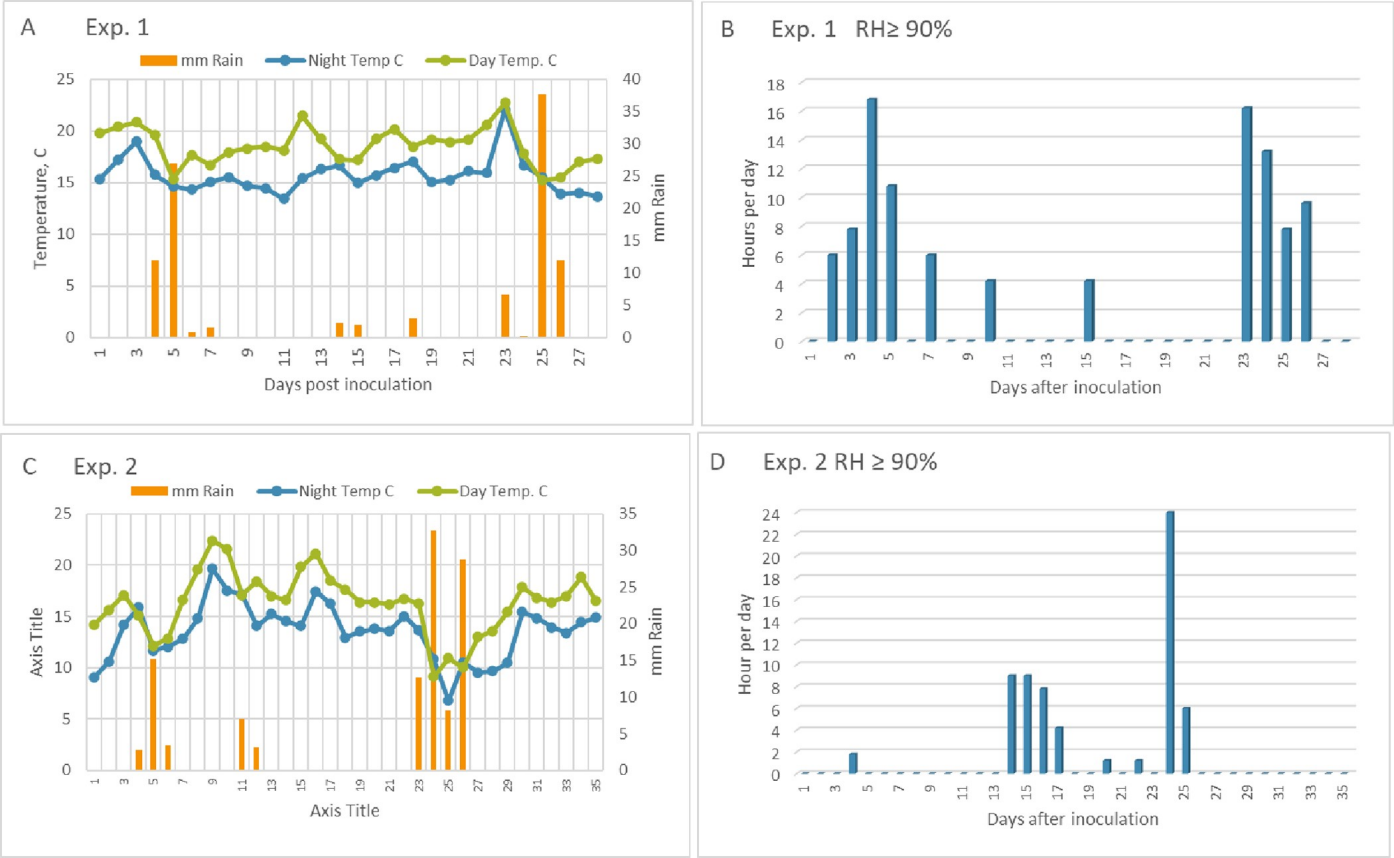
Weather conditions prevailed at BIU Farm during Exp 1 (A, B) and Exp 2 (C, D). Figures in C and D represent the number of hours per day in which RH was ≥ 90% and the temperature ranged between 9–21°C. Total number of such hours was 102.6 and 64.2 h, in Exp.1 and Exp.2, respectively.

Mean night/ day temperatures were 15.7°C/18.5°C. Mean relative humidity during the experiment was 70.3%. Ten rain events occurred during the experiment with a total rainfall accumulation of 105 mm (Fig 1A). The number of hours per day during which RH was ≥90% at air temperature of 9–21°C are shown in Fig 1B. They summed up to 102.6 hours during the experiment.

#### First symptoms

First disease symptoms appeared at 5 dpi in control untreated plots inoculated with either 23A1 or 36A2. Fungicidal spray delayed the appearance of the disease in a dose-dependent manner. Thus, in plots treated with INF, ZZ, ZF and ZE and inoculated with 23A1 disease showed up at 9–11, 12–14, 14, and 14–17 dpi, respectively (Table 1). In plots inoculated with 36A2 disease showed up at 9–11, 12, 14, and 11–15 dpi, respectively, depending on the dose applied (Table 1). Interestingly, in ZE treatments, disease appearance was delayed by 2–3 days in plots inoculated with 23A1 as compared to plots inoculated with 36A2.

**Table 1:** Exp.1. Epidemics of late blight incited by two genotypes of *P*. *infestans* in potato Nicola under field conditions during Nov-Dec 2020.

**23A1, % blighted leaf area Days post inoculation**																								
**Treatment**	**5**		**9**		**11**		**12**		**14**		**15**		**17**		**19**		**21**		**23**		**25**		**28**	
**Control**	2.10	A	4.00	A	4.00	A	15.00	A	44.00	A	50.00	A	50.00	A	59.50	A	65.00	A	78.0	A	78.0	A	94.0	A
																								
**INF 0.25**	0.00	B	0.05	B	0.50	B	8.00	B	8.30	B	20.00	B	42.50	B	52.00	B	55.00	B	55.0	B	75.0	B	77.5	B
**INF 0.5**	0.00	B	0.05	B	0.40	B	4.00	C	7.80	BC	14.00	C	17.50	C	38.50	C	41.00	C	47.0	C	75.0	B	77.5	B
**INF 1**	0.00	B	0.00	B	0.40	B	0.80	D	7.00	C	8.10	D	9.20	D	14.00	D	37.00	D	41.0	D	72.5	C	74.0	C
																								
**ZZ 0.25**	0.00	B	0.00	B	0.00	C	0.01	E	0.20	D	0.20	E	0.80	E	4.00	E	8.20	E	8.5	E	12.5	D	12.5	E
**ZZ 0.5**	0.00	B	0.00	B	0.00	C	0.00	E	0.01	D	0.06	E	0.70	E	2.50	EF	7.10	E	8.0	EF	8.5	F	9.0	FG
**ZZ 1**	0.00	B	0.00	B	0.00	C	0.00	E	0.07	D	0.07	E	0.60	E	1.20	FG	3.10	F	6.5	F	7.1	G	8.1	FG
																								
**ZF 0.25**	0.00	B	0.00	B	0.00	C	0.00	E	0.05	D	0.40	E	0.60	E	0.80	FG	0.95	G	4.0	G	11.0	E	17.0	D
**ZF 0.5**	0.00	B	0.00	B	0.00	C	0.00	E	0.03	D	0.35	E	0.50	E	0.75	FG	0.80	G	2.0	H	7.2	FG	9.5	F
**ZF 1**	0.00	B	0.00	B	0.00	C	0.00	E	0.03	D	0.16	E	0.26	E	0.50	FG	0.80	G	2.0	H	2.5	I	4.0	H
																								
**ZE 0.25**	0.00	B	0.00	B	0.00	C	0.00	E	0.05	D	0.05	E	0.20	E	0.40	FG	0.80	G	0.8	HI	8.5	F	9.0	FG
**ZE 0.5**	0.00	B	0.00	B	0.00	C	0.00	E	0.00	D	0.00	E	0.07	F	0.20	G	0.63	G	0.8	HI	4.0	H	7.5	G
**ZE 1**	0.00	B	0.00	B	0.00	C	0.00	E	0.00	D	0.00	E	0.01	F	0.05	G	0.25	G	0.3	H	0.8	J	0.9	I
**36A2, % blighted leaf area Days post inoculation**																								
**Treatment**	**5**		**9**		**11**		**12**		**14**		**15**		**17**		**19**		**21**		**23**		**25**		**28**	
**Control**	1.50	A	7.50	A	####	A	55.00	A	65.00	A	65.00	A	69.00	A	69.00	A	70.0	A	81.0	A	100.0	A	100.0	A
																								
**INF 0.25**	0.00	B	0.01	B	7.00	B	25.50	B	59.00	B	59.00	B	70.00	A	70.00	A	70.0	A	70.0	A	95.0	B	100.0	A
**INF 0.5**	0.00	B	0.03	B	4.00	C	9.70	C	51.00	C	51.00	C	59.00	B	65.00	B	68.0	A	69.0	A	92.5	C	92.5	B
**INF 1**	0.00	B	0.00	B	0.50	D	0.95	D	14.00	D	17.00	D	41.00	C	55.00	C	55.0	B	55.0	B	55.0	D	65.0	C
																								
**ZZ 0.25**	0.00	B	0.00	B	0.00	D	0.95	D	1.80	E	2.50	E	8.00	D	20.00	D	25.0	C	30.0	C	36.0	E	36.0	D
**ZZ 0.5**	0.00	B	0.00	B	0.00	D	0.12	D	0.90	E	1.10	E	4.00	E	8.10	E	17.5	D	17.0	D	20.0	F	22.5	E
**ZZ 1**	0.00	B	0.00	B	0.00	D	0.05	D	0.65	E	0.70	E	2.00	F	7.00	E	9.5	D	9.5	E	9.5	H	12.5	G
																								
**ZF 0.25**	0.00	B	0.00	B	0.00	D	0.00	D	0.20	E	0.50	E	0.80	FG	0.80	F	4.0	E	4.0	F	6.0	HI	6.5	H
**ZF 1**	0.00	B	0.00	B	0.00	D	0.00	D	0.10	E	0.20	E	0.60	FG	0.80	F	1.5	F	1.5	FG	4.0	IJ	4.0	I
																								
**ZE 0.25**	0.00	B	0.00	B	0.07	D	0.07	D	0.70	E	1.50	E	4.50	E	6.90	EF	8.5	D	9.5	F	15.0	G	17.0	F
**ZE 0.5**	0.00	B	0.00	B	0.00	D	0.00	D	0.02	E	0.12	E	0.20	G	0.70	F	0.8	F	2.1	G	3.1	IJ	6.0	HI
**ZE 1**	0.00	B	0.00	B	0.00	D	0.00	D	0.00	E	0.01	E	0.07	G	0.15	F	0.2	G	0.3	G	0.4	J	0.4	J

Potato crops were sprayed once with a fungicide, just before inoculation. Percent blighted leaf area was visually estimated at 5–28 days post inoculation. Different letters in columns indicated on a significant difference between treatments at α = 0.05.

#### Disease progress

**Fig 2:**
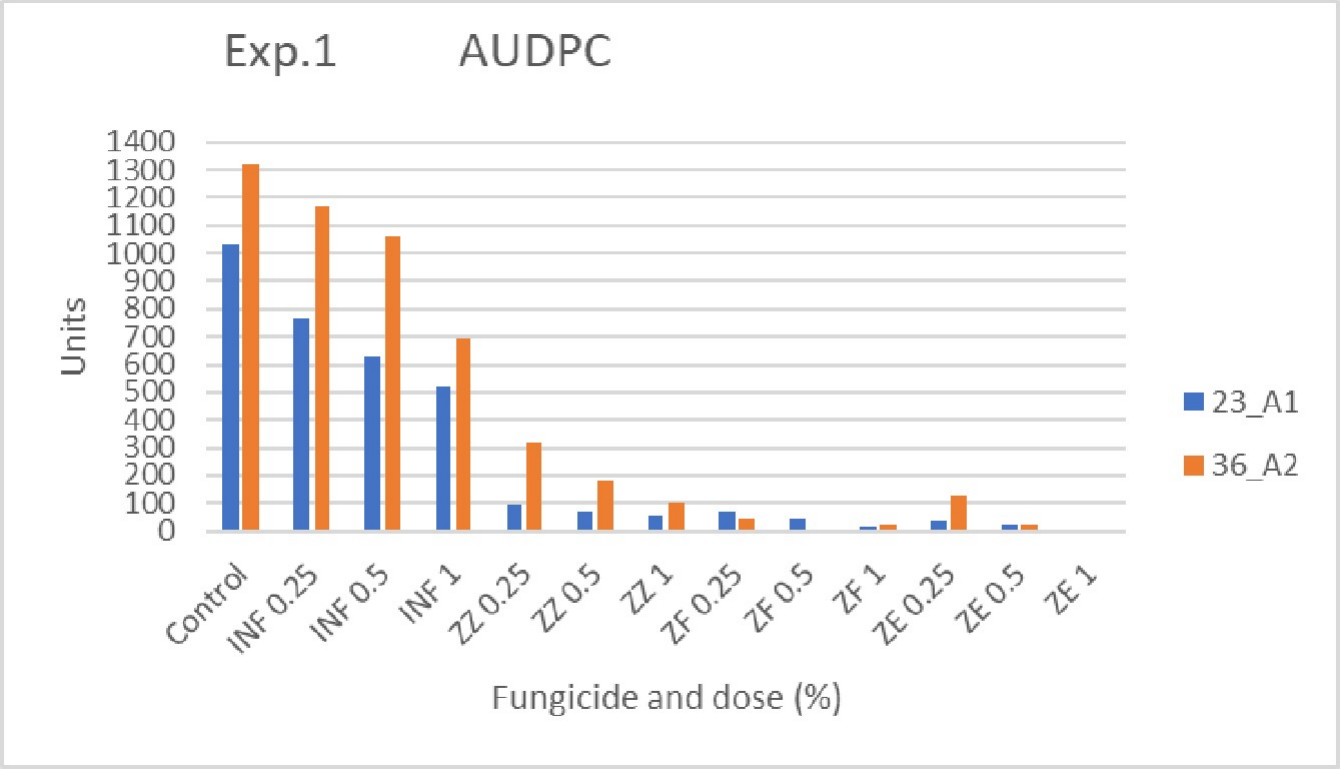
Exp 1. Area under disease progress curves (AUDPC) of late blight epidemics incited by two genotypes of *P*. *infestons* in potato Nicola under field conditions during Nov-Dec 2020. Potato crops were sprayed with a fungicide only once, just before inoculation. For statistical analysis see Table 1.

Table 1 presents disease records that were taken during the experiment, together with the statistical analysis for each scoring day. AUDPC values are illustrated in Fig 2. Disease progress was faster and final disease severity was significantly higher in control plots as compared to most fungicide-treated plots (Table 1). Among the treated plots, those treated with INF showed the fastest disease progress and highest final disease severity (Table 1). Plots treated with Zorvec mixtures were significantly less affected than the control plots (Table 1). Higher dose of a fungicide was in most cases significantly more effective in controlling the blight than a lower dose (Table 1). Zorvec-treated plots showed significantly less disease than INF-treated plots (Table 1). AUDPC values were larger for most 36A2 inoculated plots than for 23A1 inoculated plots (Fig 2).

#### Resistance monitoring

In both monitoring assays, sporulating lesions were formed in the untreated control inoculated leaves. Reduced sporulation was seen in leaves treated with oxathiapiprolin of 0.0001ppm ai. Negligible sporulation was seen in leaves treated with 0.001 ppm ai and restricted lesions with no sporulation were formed in leaves treated with 0.01 ppm ai., suggesting that no resistant mutants were produced in the field during this experiment.

### Experiment 2

#### Weather

The mean night/day temperatures were 13.5/16.3°C. Mean relative humidity during the experiment was 66.2%. Nine rain events occurred during the experiment with a total rainfall accumulation of 114 mm, (Fig 1C). The number of hours per day during which RH was ≥90% at air temperature of 9–21°C are shown in Fig 1D. They summed up to 64.2 hours during the experiment.

#### First symptoms

First disease symptoms appeared at 8 dpi in control untreated plots inoculated with either 23A1 or 36A2. In fungicide-treated plots, first lesions showed up much later. In plots treated with INF, MFX and Zorvec and inoculated with 23A1 lesions appeared at 16, 16 and 16–22 dpi, as against 16, 17 and 16–34 dpi, respectively, in plots inoculated with 36A2 (Table 2).

**Table 2:** Exp 2. Epidemics of late blight incited by two genotypes of *P*. *infestans* in potato Nicola under field conditions during Jan-Feb 2021.

**23A1, % blighted leaf area Days post inoculation**																						
**Treatment**	**8**		**14**		**16**		**17**		**20**		**22**		**24**		**27**		**29**		**31**		**34**	
**Control**	0.01	**A**	0.04	A	4.00	**A**	7.50	**B**	22.30	**B**	35.0	**A**	40.0	**A**	50.0	A	63.0	**A**	75.0	**A**	90.0	**A**
																						
**INF 0.1**	0.00	**B**	0.00	**B**	4.00	**A**	8.25	**A**	26.50	**A**	37.5	**A**	45.0	**A**	45.0	A	47.5	**B**	70.0	**A**	85.0	**A**
**INF 1**	0.00	**B**	0.00	**B**	0.34	**B**	0.65	**CD**	2.60	**C**	2.9	**B**	7.3	**B**	10.0	B	10.0	**C**	15.0	**B**	15.0	**B**
																						
**MFX 0.1**	0.00	**B**	0.00	**B**	0.06	**B**	0.75	**C**	2.30	**C**	4.0	**B**	7.5	**B**	8.0	BC	8.0	**CD**	9.75	**BC**	17.5	**B**
**MFX 1**	0.00	**B**	0.00	**B**	0.02	**B**	0.02	**CD**	0.70	**C**	2.1	**B**	5.2	**B**	9.5	B	9.5	**C**	9.25	**BCD**	14.0	**B**
																						
**ZZ 0.1**	0.00	**B**	0.00	**B**	0.02	**B**	0.01	**D**	0.38	**C**	0.4	**B**	2.0	**B**	7.2	BCD	9.75	**C**	10.1	**BC**	18.0	**B**
**ZZ 1**	0.00	**B**	0.00	**B**	0.00	**B**	0.01	**D**	0.04	**C**	0.05	**B**	0.38	**B**	1.0	CD	1.9	**DEF**	2.6	**CDE**	1.5	**C**
																						
**ZF 0.1**	0.00	**B**	0.00	**B**	0.02	**B**	0.04	**CD**	0.13	**C**	0.58	**B**	0.75	**B**	5.0	BCD	6.88	**CDE**	8.2	**CD**	9.0	**BC**
**ZF 1**	0.00	**B**	0.00	**B**	0.00	**B**	0.00	**D**	0.00	**C**	0.03	**B**	0.01	**B**	0.05	D	0.68	**EF**	0.8	**E**	0.9	**C**
																						
**ZE 0.1**	0.00	**B**	0.00	**B**	0.06	**B**	0.06	**CD**	0.14	**C**	0.28	**B**	0.65	**B**	1.9	CD	2.35	**DEF**	4.0	**CDE**	7.5	**BC**
**ZE 1**	0.00	**B**	0.00	**B**	0.00	**B**	0.00	**D**	0.00	**C**	0.01	**B**	0.01	**B**	0.03	D	0.03	**F**	0.04	**E**	0.04	**C**
**36A2, % blighted leaf area Days post inoculation**																						
**Treatment**	**8**		**14**		**16**		**17**		**20**		**22**		**24**		**27**		**29**		**31**		**34**	
**Control**	**0.01**	**A**	0.35	**A**	4.95	**A**	12.50	**A**	21.0	**B**	38.0	**A**	55.0	**A**	69.0	A	72.5	**A**	76.5	**A**	94.0	**A**
																						
**INF 0.1**	0.00	**B**	0.00	**B**	1.10	**B**	8.10	**B**	30.0	**A**	40.0	**A**	51.0	**B**	51.0	B	52.0	**B**	73.0	**B**	81.5	**B**
**INF 1**	0.00	**B**	0.00	**B**	0.02	**C**	0.46	**C**	6.0	**C**	6.9	**B**	9.6	**C**	9.6	C	9.8	**C**	12.5	**C**	23.0	**C**
																						
**MFX 0.1**	0.00	**B**	0.00	**B**	0.00	**C**	0.05	**C**	0.65	**D**	0.85	**C**	1.9	**D**	2.9	D	3.0	**E**	2.4	**EF**	4.0	**E**
**MFX 1**	0.00	**B**	0.00	**B**	0.00	**C**	0.04	**C**	0.6	**D**	1.68	**C**	1.0	**D**	3.1	D	3.0	**E**	3.0	**E**	4.0	**E**
																						
**ZZ 0.1**	0.00	**B**	0.00	**B**	0.04	**C**	0.13	**C**	0.6	**D**	0.75	**C**	2.0	**D**	5.0	D	7.1	**D**	7.5	**D**	9.0	**D**
**ZZ 1**	0.00	**B**	0.00	**B**	0.01	**C**	0.00	**C**	0.02	**D**	0.03	**C**	0.04	**D**	0.11	E	0.03	**E**	0.40	**FG**	0.5	**F**
																						
**ZF 0.1**	0.00	**B**	0.00	**B**	0.00	**C**	0.01	**C**	0.01	**D**	0.03	**C**	0.03	**D**	0.07	E	0.11	**E**	1.05	**EFG**	6.8	**DE**
**ZF 1**	0.00	**B**	0.00	**B**	0.00	**C**	0.00	**C**	0.01	**D**	0.05	**C**	0.05	**D**	0.05	E	0.05	**E**	0.10	**FG**	0.02	**F**
																						
**ZE 0.1**	0.00	**B**	0.00	**B**	0.00	**C**	0.00	**C**	0.03	**D**	0.04	**C**	0.05	**D**	0.01	E	0.02	**E**	0.25	**FG**	0.40	**F**
**ZE 1**	0.00	**B**	0.00	**B**	0.00	**C**	0.00	**C**	0.00	**D**	0.00	**C**	0.00	**D**	0.00	E	0.00	**E**	0.00	**G**	0.05	**F**

Potato crops were sprayed once with a fungicide, just before inoculation. Percent blighted leaf area was visually estimated at 8–34 days post inoculation. Different letters in columns indicated on a significant difference between treatments at α = 0.05.

#### Disease progress

**Fig 3:**
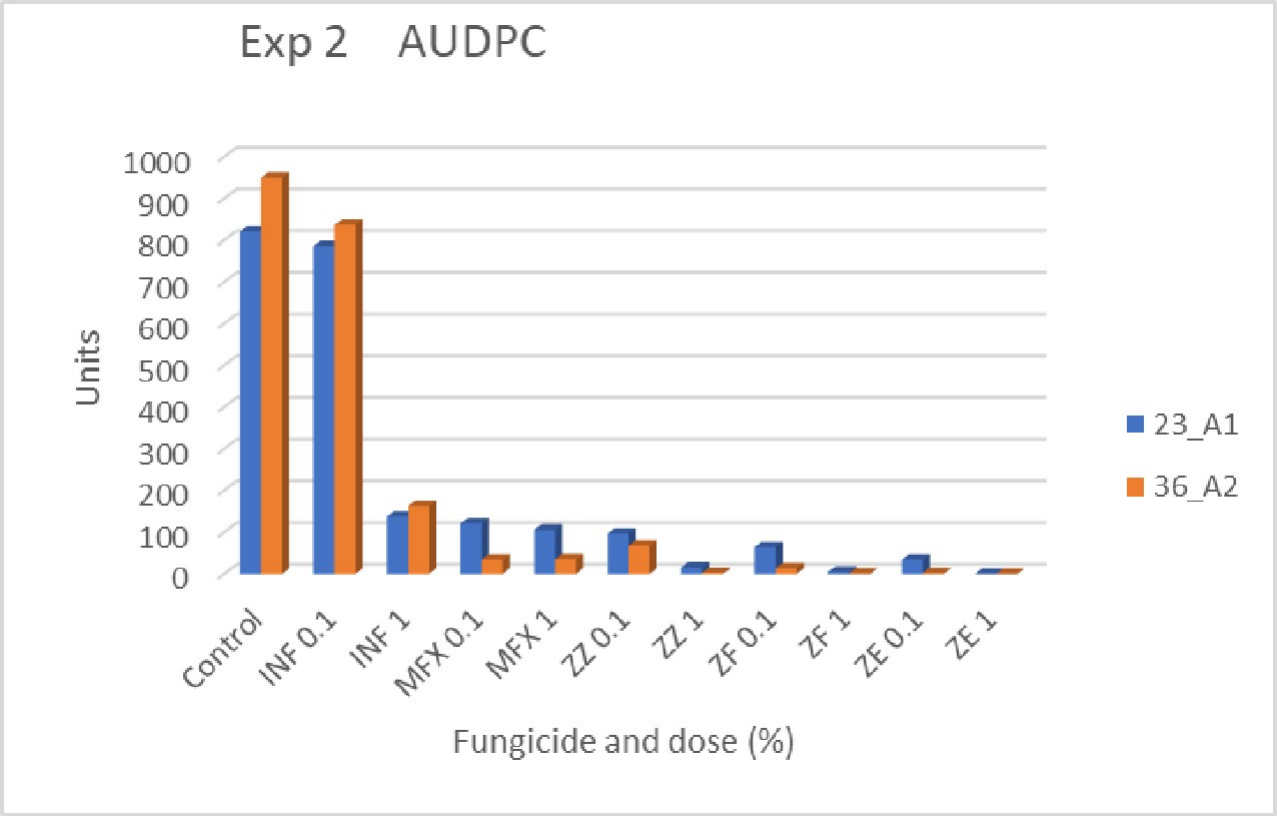
Exp.2. Area under disease progress curves (AUDPC) of late blight epidemics incited by two genotypes of *P*. *infestons* in potato Nicola under field conditions during Jan-Feb 2021. Potato crops were sprayed with a fungicide only once, just before inoculation. For statistical analysis see Table 2.

Table 2 presents disease records that were taken during the experiment, together with the statistical analysis for each scoring day. AUDPC values are illustrated in Fig 3. The fastest progress of the disease occurred in control inoculated plots and in plots treated with 0.1% INF. Moderate progress was recorded in 1% INF and MFX treated plots. The slowest progress occurred in Zorvec treated plots. ZE was best performing among the three Zorvec mixtures (Table 2). At the end of the experiment, the mean percent blighted foliage area in control plots inoculated with 23A1 and 36A2 was 90% and 94%, respectively (Table 2). Plots treated with a fungicide (except INF of 0.1% with 23A1) were all significantly less affected than the control plots (Table 2). The least affected plots were those treated with ZE of 1% (Table 2). AUDPC values were larger for most fungicide-treated 23A1 inoculated plots than for fungicide-treated 36A2 inoculated plots (Fig 3).

#### Resistance monitoring

Results in Exp 2 were like those obtained with Exp.1 (see above), suggesting that no resistant mutants were produced in the field during this experiment.

#### Compared efficacy of oxathiapiprolin mixtures in Exp 1 and Exp 2

Data in Table 3 present the comparative efficacy in both experiments of ZZ, ZF and ZE in controlling 23A1 and 36A2. All three mixtures were significanly more effective at a higher than a lower dose, except ZE in Exp 2. Disease records in Table 3 indicate that efficacy ranked in the order ZE > ZF > ZZ. In Exp 1, ZE of 1% provided significantly better control of both 23A1 and 36A2 than ZF or ZZ of the same dose. In Exp 2, it was true for 0.1% but not for 1%.

**Table 3:** Comparative efficacy of three oxathiapiprolin fungicidal mixtures in controlling two genotypes of *P*. *infestans* in two field experiments.

Exp 1	% blighted leaf area, 28 dpi				Exp 2	% blighted leaf area, 34 dpi			
Treatment	23A1	23A1	36A2	36A2	Treatment	23A1	23A1	36A2	36A2
**ZZ 0.25**	12.5	B	36	A	**ZZ 0.1**	18	A	9	A
**ZZ 0.5**	9	CD	22.5	B					
**ZZ 1**	8.1	CD	12.5	D	**ZZ 1**	1.5	C	0.5	C
**ZF 0.25**	17	A	6.5	E	**ZF 0.1**	9	B	6.8	B
**ZF 0.5**	9.5	C	no data						
**ZF 1**	4	E	4	F	**ZF 1**	0.9	C	0.2	C
**ZE 0.25**	9	CD	17	C	**ZE 0.1**	7.5	B	0.4	C
**ZE 0.5**	7.5	D	6	E					
**ZE 1**	0.9	F	0.4	G	**ZE 1**	0.04	C	0.05	C

The figures present the final disease score at the end of each experiment. Different letters in columns, in each experiment, indicate on a significant difference between treatments in each experiment at α = 0.05.

## Discussion

Mefenoxam and Infinito are among the most widely used fungicides in Israel for the control of late blight in potato. Resistance to mefenoxam appeared in the country in 1983 [14,15]. However, the proportion of resistant isolates in the population fluctuated between seasons and within seasons which made its use possible when sensitive isolates prevailed [13–15]. Infinito performed well against late blight in recent years but loss of efficacy was noticed in the last season [U. Zig, *personal communication*].

Zorvec mixtures were recently introduced to the market. The efficacy of Zorvec Endavia (oxathiapiprolin+ benthiavalicarb = ZE) against late blight was recently reported [8] but no reports are available on the efficacy of Zorvec Vinabel (oxathiapiprolin+ zoxamide = ZZ) or Zorvec Encantia (oxathiapiprolin+ famoxadone = ZF).

The purpose of the present study was to re-evaluate the efficacy of Mefenoxam and Infinito and to measure the efficacy over time of three newly introduced Zorvec mixtures against late blight in potato. Two experiments were conducted in the field, in two consecutive seasons. The potato crops were sprayed only once with 2 or 3 doses of 5 fungicides and thereafter artificially inoculated with two genotypes (lineages) of the pathogen which have prevailed in the country last year. Monitoring the resistance against oxathiapiprolin was followed during the experiments.

Weather conditions were more conducive for late blight development in Exp 1 than in Exp 2. Night/day temperatures were more optimal in Exp. 1 (15.7/18.5°C) as compared to Exp.2 (13.5/16.3°C). Total rain fall was similar (105 and 114 mm in Exp 1 and Exp 2, respectively) but the number of hours with RH of ≥90% was 59% higher in Exp 1 than in Exp 2 (102.6 as against 64.2). These differences in the weather conditions may be partly responsible for the enhanced pathogenicity of genotype 36A2 in Exp 1 but not in Exp 2. It might occur that 36A2 can better proliferate under optimal conditions while 23A1 can do it under suboptimal conditions. Genotype 36A2 is a newcomer to Israel, arrived from Europe in seed tubers in 2019 [13]. Genotype has 23A1 occurred in Israel since 2004 and, from 2010 has dominated the population in most years [13]. During the spring season of 2020, the Israeli population of *P*. *infestans* (80 isolates) consisted of 23A1: 36A2 at a ratio of 1:1 (D.L. Cooke, *personal communication*). We selected 15 isolates of each genotype to conduct the present experiments.

The fact that the fungicides were applied only once allowed us to determine the duration of full protection that each fungicide could provide (days until first lesions appeared) and to follow their efficacy overtime. This information is crucial to determine the interval periods between sprays and the number of sprays per season required of each fungicide and dose.

The data obtained from both experiments teach that INF was poorly effective against either genotype of the pathogen whereas Zorvec was highly effective against both. Mefenoxam was partly effective, more so against 36A2 as this lineage was composed of mainly Mefenoxam-sensitive isolates.

Zorvec mixtures provided excellent control of both genotypes of the pathogen. They delayed the onset of the disease and greatly suppressed its progress. ZE exceeded ZF and ZZ in both parameters (onset and progress of the disease) in both experiments as evident in Tables 1–3. These attributes of ZE have probably derived from the systemic nature of the partner fungicide benthiavalicarb [16].

Monitoring data revealed no buildup of resistance against oxathiapiprolin in either experiment in-spite of the fact that its doses exceeded the recommend dose.

Our data suggest that fungicidal control efficacy of late blight in the field depends not only on the chemistry of the fungicide and its dose but also on the genotype of the pathogen prevailing in the field. The fungicide Zorvec Encantia (= ZE), made of oxathiapiprolin and benthiavalicarb, was proven to be highly effective against the two lineages of *P*. *infestans* used in this study. A single application of 0.1% ZE provided, at the end of the season, 91.2% and 99.6% protection (% decrease in AUDPC in treated plots relative to AUDPC in control untreated plots) against 23A1 and 36A2, respectively. This conclusion is further supported by another experiment (not shown) in which 0.1% ZF or 0.1% ZE, each applied twice to potato crops at 10 days interval, provided at the end of the season protection of 82.5% and 99.3%, respectively.
